# Polyester Sulphonic Acid Interstitial Nanocomposite Platform for Peroxide Biosensor

**DOI:** 10.3390/s91209965

**Published:** 2009-12-08

**Authors:** Amir Al-Ahmed, Peter M. Ndangili, Nazeem Jahed, Priscilla G. L. Baker, Emmanuel I. Iwuoha

**Affiliations:** SensorLab, Department of Chemistry, University of Western Cape, Bellville, 7535, Cape Town, South Africa; E-Mails: aal-ahmed@uwc.ac.za (A.A.-A.); pndangili@uwc.ac.za (P.M.N.); njahed@uwc.ac.za (N.J.); eiwuoha@uwc.ac.za (E.I.I.)

**Keywords:** peroxide biosensors, immobilization of enzyme, HRP, electrochemical polymerisation, polyaniline nanotubes, polyester sulphonic acid

## Abstract

A novel enzyme immobilization platform was prepared on a platinum disk working electrode by polymerizing aniline inside the interstitial pores of polyester sulphonic acid sodium salt (PESA). Scanning electron microscopy study showed the formation of homogeneous sulphonated polyaniline (PANI) nanotubes (∼90 nm) and thermogravimetric analysis (TGA) confirmed that the nanotubes were stable up to 230 °C. The PANI:PESA nanocomposite showed a quasi-reversible redox behaviour in phosphate buffer saline. Horseradish peroxidase (HRP) was immobilized on to this modified electrode for hydrogen peroxide detection. The biosensor gave a sensitivity of 1.33 μA (μM)^-1^ and a detection limit of 0.185 μM for H_2_O_2_. Stability experiments showed that the biosensor retained more than 64% of its initial sensitivity over four days of storage at 4 °C.

## 1. Introduction

Among the electrically conducting polymers, polyaniline (PANI) presents the best combination of stability, conductivity and easy procedure for its synthesis [1-5]. As a consequence PANI, its derivatives, as well as its conducting composites are extensively used in biosensors and bioelectrochemical switches [5,6]. The structure of PANI is affected by the conditions employed during synthesis, such as the concentration of monomer, pH of the electrolyte, presence of additives, nature of doping agent and, in the case of electrosynthesis, the applied electrode potential [1-6]. The conductivity of PANI films depends on the degree of protonation along the polymer back-bone. This was a major problem for its application in biosensors which require neutral or slightly alkaline media for their operation. One approach that was developed for solving the problem was the doping of PANI with polystyrene sulphonic acid (PSS) and polyvinylsulphonate (PVS) [5-9]. Many biosensors have been reported in which PANI and its derivatives were used as immobilisation layers [6-9], either alone or in combination with glutaraldehyde or polymeric materials such as nafion and Eastman AQ polymers [9-13]. These biosensors are characterised by excellent properties in terms of reproducibility, response time and storage stability.

In this work a PANI nanocomposite was prepared with aniline and polyester sulphonic acid sodium salt. The precursor aniline-PESA mixture (14:1 w/w) was chemically polymerised on a Pt disk working electrode in HCl medium using (NH_4_)_2_S_2_O_8_ as oxidant. The resultant PANI-PESA nanocomposite was used in peroxide biosensor fabrication.

## 2. Experimental

### 2.1. Reagents

Aniline, ammonium persulphate, hydrochloric acid, horseradish peroxidase 160 units/mg, polyvinyl sulphonate (25% solution in water) and hydrogen peroxide (30% solution) were supplied by Sigma Aldrich. Linear polyester sulphonic acid sodium salt or Eastman AQ polymer 55S (here '55′ stands for the glass transition temperature and ‘S’ stands for solid physical state of the polymer) was purchased from Eastman Chemical B.V. South Africa Ltd. All solutions were prepared with water purified with a Millipore water-purification apparatus to a final conductivity of 18.2 Ω.

### 2.2. Instrumentation

Electrochemical experiments were carried out using a BAS 50 automated electrochemical workstation from Bio-Analytical Systems (BAS, USA) and a 1.6 mm diameter Pt disk working electrode, Pt wire auxiliary electrode and Ag/AgCl reference electrode. A Joel Scanning Electron Microscope (JSM 7500F) was used to study the surface morphology of the composite materials. FTIR spectra of the pure and composite materials were obtained with a Perkin Elmer Spectrum 100 series instrument. Thermogravimetric analysis (TGA) was performed on a Perkin Elmer TGA7 instrument under nitrogen at a heating rate of 10 °C/min.

### 2.3. Fabrication of Biosensor

The PANI-PESA composite material was prepared from a mixture consisting of 10.3 mg aniline and 0.7 mg PESA (*i.e.*, 14:1 w/w aniline/PESA ratio). 6 μL of this mixture was placed on a clean Pt disk electrode and allowed to dry. The electrode was placed upside down inside a glass vial in such a way that the Pt surface containing the mixture dipped into a 10 mL solution containing 0.1 M (NH_4_)_2_S_2_O_8_ solution and 300 μL PVS in 1 M HCl. Aniline was polymerized inside the interstitial pores of PESA matrix using (NH_4_)_2_S_2_O_8_ as oxidant. The polymerization process was allowed to proceed for 12 h in an ice bath. After polymerization a thick layer of the nanocomposite material was formed on the Pt electrode surface. The electrode was sequentially washed with 1 M HCl, distilled water and 0.1 M phosphate buffer pH 6.5. Electrochemical properties of the modified electrode were studied in 1 M HCl and in 0.1 M phosphate buffer using cyclic voltammetry (CV) and square wave voltammetry (SWV). Freshly synthesized composite material was carefully removed from the electrode surface and then characterized by FTIR, SEM and TGA.

Horseradish peroxidase was immobilized on the Pt/PANI:PESA electrode by electrostatic attachment technique. Here Pt/PANI:PESA was reduced for 15 min in 1 mL 0.1 M phosphate buffer pH 6.5 at a constant potential of -500 mV. Then the Pt/PANI:PESA electrode was placed in a cell containing 100 μL HRP solution (10 mg/mL HRP, 3 mg BSA) and 900 μL 0.1 M phosphate buffer pH 6.5 and oxidized for 20 min at a potential of +650 mV. During the oxidation process HRP was electrostatically attached to the nanocomposite layer [14,15] to form Pt/PANI:PESA/HRP biosensor. The bioelectrode was then rinsed with distilled water to remove unattached enzymes. To select the optimum pH for operating the biosensor, five 1 mM H_2_O_2_ solutions were prepared with 0.1 M phosphate buffer solutions of pH 4, 6.5, 7, 8 and 10. The CV and SWV responses of the biosensor at the five pH's were determined using 3 mL of the H_2_O_2_ solutions. Maximum response of the biosensor was obtained with 0.1 M phosphate buffer at pH 6.5. All biosensor experiments were then performed at pH 6.5.

### 2.4. Biosensor Response

Cyclic and square wave voltammetric responses of the biosensor were recorded by successively adding 3 μL aliquots of 1 mM H_2_O_2_ to a 1 mL cell solution containing 0.1 M phosphate buffer at pH 6.5. The long term stability of the bioelectrode was investigated by evaluating the changes in the biosensor response to H_2_O_2_ with time. In this case freshly prepared biosensor was placed in an electrochemical cell containing 3 mL of phosphate buffer and aliquots of 3 μL of 1 mM H_2_O_2_ were added successively under argon atmosphere in a stirred solution at 21 ± 2 °C. The bioelectrode was stored in phosphate buffer at 4 °C when not in use. The experiment was repeated with the same electrode every 36 h.

## 3. Results and Discussion

### 3.1. Characterization of PANI:PESA Composite

**Figure 1.**
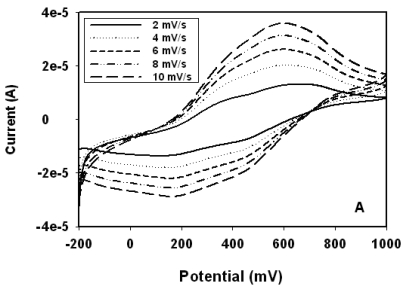

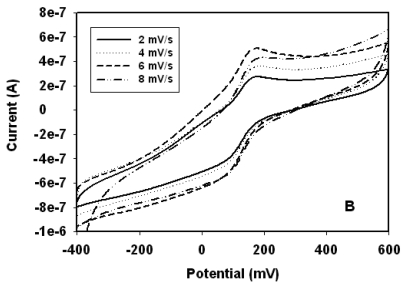
CV of PANI:PESA nanocomposite in (A) 1 M HCl and (B) in 0.1 M phosphate buffer (pH 6.5).

**Figure 2.**
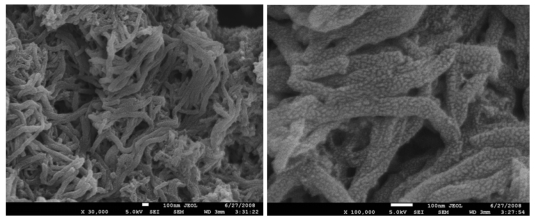
SEM image of PANI:PESA showing ∼90 nm (diameter) nanotubes.

The loading of the nanocomposite on the electrode surface was gravimetrically determined and it was found that ∼3.3 mg of the PANI:PESA composite material was deposited on the electrode surface. Figure 1(A) shows the low scan-rate CV's of PANI:PESA in 1 M HCl. The CV shows tow main redox couples at a scan rate of 2 mV/s, corresponding to leucoemeraldine/leucoemeraldine radical cation (200 mV/350 mV) and pernigraniline/pernigraniline radical action (600 mV/470 mV) transitions [7,11,16]. However, as the scan rate increases the fully reduced and oxidised forms of the polymer, leucoemeraldine (200 mV) and pernigraniline (600 mV), become more prominent. This shows that the formation of the radical cations are slow electron transfer processes. In buffer medium (Figure 2B) only the electrochemistry of the leucoemeraldine/leucoemeraldine radical cation (10 mV/100 mV) redox couple was observed. This behaviour of the composite electrode in buffer medium indicates that strong acidic conditions are required for the oxidation of the PANI composite to the pernigraniline form. The CV of the PANI:PESA electrode in buffer gave peak separation, ΔE_p_, value of 70 mV ± 6 mV at all scan rates and the peak potential, E_p_, values were independent of scan rate. This is as expected for the quasi-reversible electrochemistry of surface-bound electroactive species [11,17]. This behaviour of the polymeric material in buffer together with the associated low redox potential make the PANI-PESA composite suitable as a platform for biosensor preparation.

**Figure 3.**
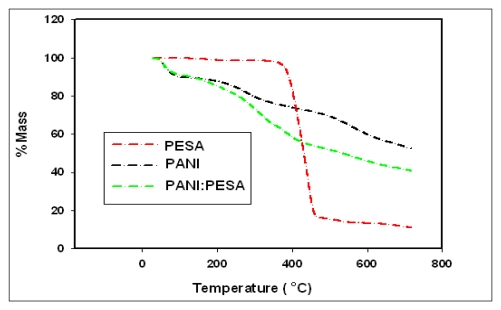
TGA plots of PESA, PANI and PANI:PESA.

**Figure 4.**
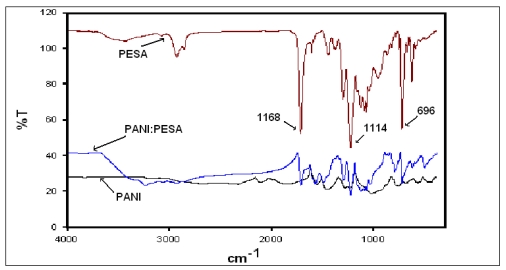
FTIR spectra of PESA, PANI and PANI:PESA.

SEM images of PANI-PESA composite material are shown in Figure 2. Surface morphology showed homogeneity and formation of uniform nanotubes of ∼90 nm in diameter. The TGA curve of PANI-PESA presented in Figure 3, shows that this material is stable up to 230 °C and retains up to 80% of its initial weight till 250 °C. FTIR spectra in Figure 4 show that PANI-PESA nanocomposite material displayed all the characteristic peaks for polyaniline [18]. The bands corresponding to the stretching vibrations of N-B-N and N=Q=N structure appeared at 1501 cm^-1^ and 1573 cm^-1^, respectively (-B- and =Q= stand for benzenoid and quinoid moieties in the polyaniline backbone). The small band corresponding to the vibration mode of N=Q=N ring appeared at 1152 cm^-1^, and the stretching mode of C-N gave a band at 1294 cm^-1^.

### 3.2. pH Response of Pt/PANI-PESA/HRP

**Figure 5.**
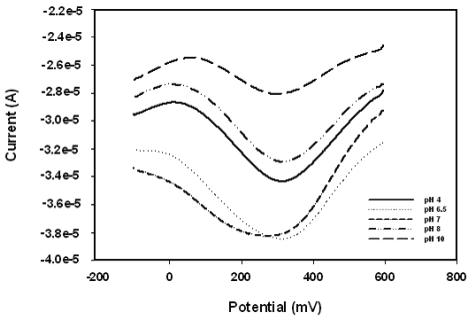
The pH dependence of the SWV responses of the biosensor to 1 mM H_2_O_2_.

**Figure 6.**
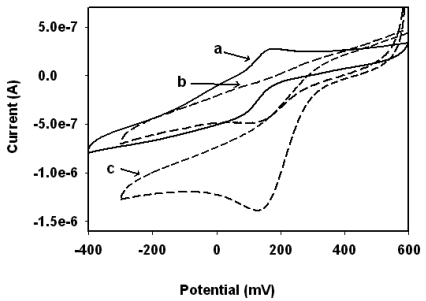
Cyclic voltammograms of (a) Pt/PANI:PESA, (b) Pt/PANI:PESA/HRP and (c) Pt/PANI:PESA/HRP/11.85 μM H_2_O_2_ in 0.1 M phosphate buffer pH 6.5 at 2 mV/s.

A freshly prepared enzyme electrode was used to check the pH dependence of the responses of the enzyme electrode to a fixed concentration of H_2_O_2_. The sensor gave a maximum response at pH 6.5 (Figure 5). This result is similar to that reported by Chen *et al*. [13-15]. Thus phosphate buffer pH 6.5 was used for all the biosensor-related studies. In Figure 6 the cyclic voltammograms of Pt/PANI:PESA, Pt/PANI:PESA/HRP and Pt/PANI:PESA/HRP/11.85 μM H_2_O_2_ are presented. The Pt/PANI:PESA electrode gave a quasi-reversible redox response in phosphate buffer pH 6.5. But when HRP was immobilised on this nanocomposite surface the enzyme electrode gave irreversible electrochemical response with a +70 mV anodic shift in the cathodic peak potentials. This shows that the electron transfer process of PANI-PESA is coupled to catalytic processes involving HRP.

### 3.3. Biosensor Response to H_2_O_2_

**Figure 7.**
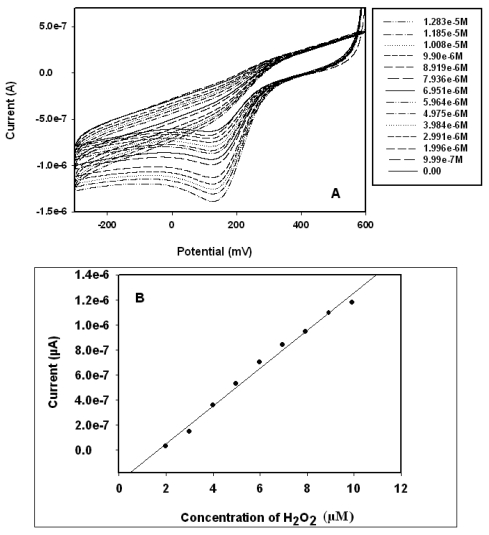
(A) Cyclic voltammograms of the biosensor responses to H_2_O_2_ in 0.1 M phosphate buffer pH 6.5; (B) Calibration plot of the biosensor for H_2_O_2_.

The electrocatalytic response of Pt/PANI:PESA/HRP biosensor to H_2_O_2_ was investigated in 0.1 M phosphate buffer pH 6.5 by cyclic voltammetry and square wave voltammetry. The cyclic voltammograms are presented in Figure 7(A). Upon the addition of H_2_O_2_ to the electrochemical cell, the reduction peak current of the biosensor increased, indicating electrocatalytic reduction of H_2_O_2_ at the HRP electrode. In this study, the first step in the catalytic cycle involves a reaction between H_2_O_2_ and Fe^3+^ of HRP (the resting state of HRP-Fe(III)). This reaction forms HRP-Fe^+•^(IV)-O, which is a high oxidation state intermediate comprising an Fe^4+^ oxoferryl centre and a porphyrin-based radical cation. Then this HRP-Fe^+•^(IV)-O reacts with PANI:PESA(red) to form HRP-Fe^••^(IV)-OH and PANI:PESA(oxid-I). The HRP-Fe^+•^(IV)-OH comprises the same oxidation state as HRP-Fe^+•^(IV)-O, that is, one oxidation state above the resting state of HRP. Another one-electron transfer reaction returns HRP-Fe^••^(IV)-OH to the resting state of HRP *i.e.*, HRP-Fe(III), while PANI:PESA(oxid-I) is oxidized to PANI:PESA(oxid-II) sate. This PANI:PESA(oxid-II) undergoes a two-electron reduction on Pt electrode to give its reduced form, PANI:PESA (red). This reaction scheme is summarised below.

(1) $$\mathrm{HRP}-\mathrm{Fe}(\mathrm{III})+H_{2}O_{2}\to \mathrm{HRP}-{\mathrm{Fe}}^{+•}(\mathrm{IV})-O+2H_{2}O$$

(2) $$\mathrm{HRP}-{\mathrm{Fe}}^{+•}(\mathrm{IV})-O+\mathrm{Pt}/\mathrm{PANI}:\mathrm{PESA}(\mathrm{red})\to \mathrm{HRP}-{\mathrm{Fe}}^{••}(\mathrm{IV})-\mathrm{OH}+\mathrm{Pt}/\mathrm{PANI}:\mathrm{PESA}(\mathrm{oxid}-I)$$

(3) $$\text{HRP}-{\mathrm{Fe}}^{••}(\text{IV})-\mathrm{OH}+\text{Pt}/\text{PANI}:\text{PESA}(\text{oxid}-I)\to \mathrm{HRP}-\text{Fe}(\text{III})+\mathrm{Pt}/\mathrm{PANI}:\mathrm{PESA}(\mathrm{oxid}-\text{II})$$

(4) $$\mathrm{Pt}/\mathrm{PANI}:\text{PESA}(\mathrm{Oxid}-\mathrm{II})+2e^{-}\to \mathrm{Pt}/\mathrm{PANI}:\mathrm{PESA}(\text{red})$$

The calibration curve of the hydrogen peroxide biosensor is shown in Figure 7(B). The values plotted were the dependence of the cathodic peak currents of the CVs in Figure 7(A) on H_2_O_2_ concentration. The figure shows a linear range of 1.99–9.90 μM H_2_O_2_. The detection limit and sensitivity of the biosensor were calculated to be 0.185 μM and 1.33 μA (μM)^-1^, respectively. The detection limit and sensitivity values obtained from this experiment compare very well with literature values. For example Chen *et al*. reported [21-24] a detection limit of 0.93 μM and sensitivity 2.42 μA (μM^-1^). The sensitivity value of Chen *et al*. in units of μA cm^-2^ (μM^-1^) was converted to μA (μM^-1^) using the surface area value of the Pt electrode used in our study, *i.e.*, 2.0106 × 10^-2^ cm^-2^. Therefore, this nanocomposite may offer a suitable alternative substrate layer for enzyme immobilization in biosensor fabrication [25]. A control experiment performed with Pt/PANI:PESA only (*i.e.*, without immobilizing any enzyme on the electrode surface) by keeping all other parameter as in the experiment with the biosensor, and no increase in reduction current was observed either with CV or SWV. This confirmed that the increase in the CV and SWV cathodic currents that were observed in the biosensing reaction involving Pt/PANI:PESA/HRP electrode is solely due to the catalytic reduction of H_2_O_2_ by HRP.

### 3.4. Stability of the Biosensor

**Figure 8.**
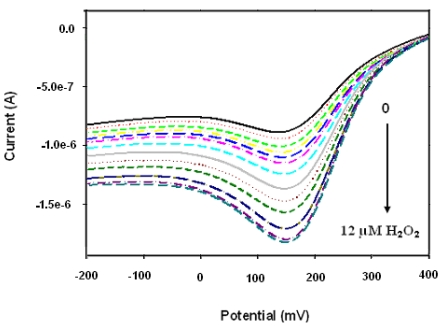
Square wave responses of Pt/PANI:PESA/HRP biosensor on the first day of biosensor preparation, *i.e.*, at 0 h.

**Figure 9.**
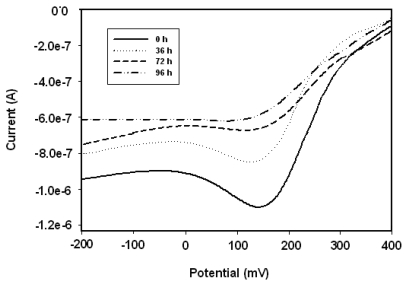
Rate of change of square wave responses of the Pt/PANI:PESA/HRP biosensor to 5.0 μM H_2_O_2_.

Square wave voltammograms presented in Figure 8 show the responses of the biosensor to hydrogen peroxide on the first day of biosensor preparation (*i.e.*, at 0 h). The sensitivity values calculated from the calibration plots of the biosensor are 1.33, 0.931 and 0.855 μA (μM^-1^) for 0 h, 36 h and 72 h, respectively. The biosensor retains more than 64% of its initial sensitivity, even after 72 h. The decrease in the electrocatalytic response with increasing storage time, as well as the decrease in sensitivity, are attributable to the denaturation of HRP on the nancomposite layer and some leaching of the enzyme from the electrode surface while stored at 4 °C in PBS. The rate of change of the cathodic response of the sensor with time is given in Figure 9 for 5.0 μM of H_2_O_2_.

## 4. Conclusions

Polyaniline nanotubes were electrosynthesised in the interstitial pores of a layer of PESA adsorbed on Pt disk electrode. The nanocomposite gave reversible electrochemistry and was tested as possible platform for biosensor by the immobilisation of HRP on the polymer matrix. The peroxide biosensor exhibited sensitivity, limit of detection and dynamic linear range characteristics for hydrogen peroxide that compare favourably with those reported by other worker.
